# Von Hipple-Lindau disease complicated with central retinal vein occlusion: a case report

**DOI:** 10.1186/s12886-022-02661-y

**Published:** 2022-11-16

**Authors:** Xingwang Chen, Mengyao Wang, Yuan Tang, Bing Xie, Xiaomei Nie, Shanjun Cai

**Affiliations:** 1 Department of Ophthalmology, Affiliated Hospital of Zunyi Medical University, No. 149, Dalian Road, Zunyi, 563000 Guizhou Province China; 2 Guizhou Eye Hospital, Zunyi, China; 3 Guizhou Provincial Branch of National Eye Disease Clinical Research Center, Zunyi, China; 4 Special Key Laboratory of Ocular Diseases of Guizhou Province, Zunyi Medical University, Zunyi, China; 5 Chongqing Aier General Hospital, Chongqing, China; 6 Guiyang Aier Eye Hospital, Guiyang, China; 7 Department of Ophthalmology, The Second Affiliated Hospital of Zunyi Medical University, Zunyi, China

**Keywords:** von Hipple-Lindau disease, Central retinal vein occlusion, Polycythemia, *VHL* gene mutation, Case report

## Abstract

**Background:**

Central Retinal Vein Occlusion (CRVO) is a rare complication of von Hipple-Lindau (VHL) disease. This report presents the first case of VHL disease complicated with CRVO caused by *VHL* c.208G > A mutation.

**Case presentation:**

A 20 s man whose left eye visual acuity gradually declined for half a year. The visual acuity of the left eye is counting fingers. Fundus examination revealed that retinal hemangioblastoma was also found in addition to typical CRVO signs such as tortuous expansion of retinal veins and flame-shaped hemorrhage of the retina. Liver tumor, cerebral infarction and erythrocytosis were found during systemic examination, and the diagnosis of polycythemia was confirmed by bone marrow smear. Furthermore, both family history and genetic analysis indicated that the patient had VHL disease caused by *VHL* c.208G > A. In this patient, a large number of bone marrow erythrocytes proliferated due to VHL disease, which led to the increase of blood viscosity and erythrocyte vascular adhesion, resulting in the obstruction of central retinal vein blood flow, and finally CRVO. For CRVO and its pathogenic factor polycythemia, patient received laser retinal photocoagulation and phlebotomies. After a 1-year follow-up, the vision in the left eye improved to 0.2 logMAR.

**Conclusions:**

This is a rare case of polycythemia complicated by CRVO in patient with VHL disease. It reminds us that the systemic disease factors should be fully considered in the diagnosis of young patients with CRVO, and that treatment requires a coordinated effort of physicians.

## Background

von Hippel-Lindau (VHL) disease is an autosomal dominantly inherited tumor syndrome, which is caused by mutations of the *VHL* gene. The *VHL* gene is located on the short arm of chromosome 3 and encodes a tumor suppressor. The *VHL* gene encodes the VHL protein (pVHL), which is a tumor suppressor. The pVHL combines with elongation factors B, C, and Cullin-2 to form E3 ubiquitin ligase. The compound can mediate the degradation of HIFα and is a key component of the oxygen sensing pathway. Mutations in the pVHL can cause HIF-dependent and HIF-independent effects leading to VHL disease. More than 500 *VHL* gene mutations related to the disease have been reported [1]. And these different mutations were associated with the different clinical phenotypes [2]. VHL disease is characterized by multiorgan and multicenter tumors, such as central nervous system hemangioblastoma (CHB), retinal hemangioblastoma (RHB), renal cell carcinoma (RCC), renal cysts, pancreatic tumor, pheochromocytoma, endolymphatic-sac tumor, and papillary cystadenoma [1]. Clinically, patients are divided into type 1 and type 2 according to whether they have pheochromocytoma [3]. CHB, RCC, RHB, pancreatic tumor, and pheochromocytoma are the most common symptoms of VHL disease [4]. And, less than 20% of VHL disease patients present with polycythemia [5]. However, VHL disease-related ocular ischemic issues have rarely been described and discussed. In this study, we describe a case of VHL disease complicated with central retinal vein occlusion (CRVO), an ocular ischemia condition that was caused by secondary polycythemia.

## Case presentation

**Fig. 1:**
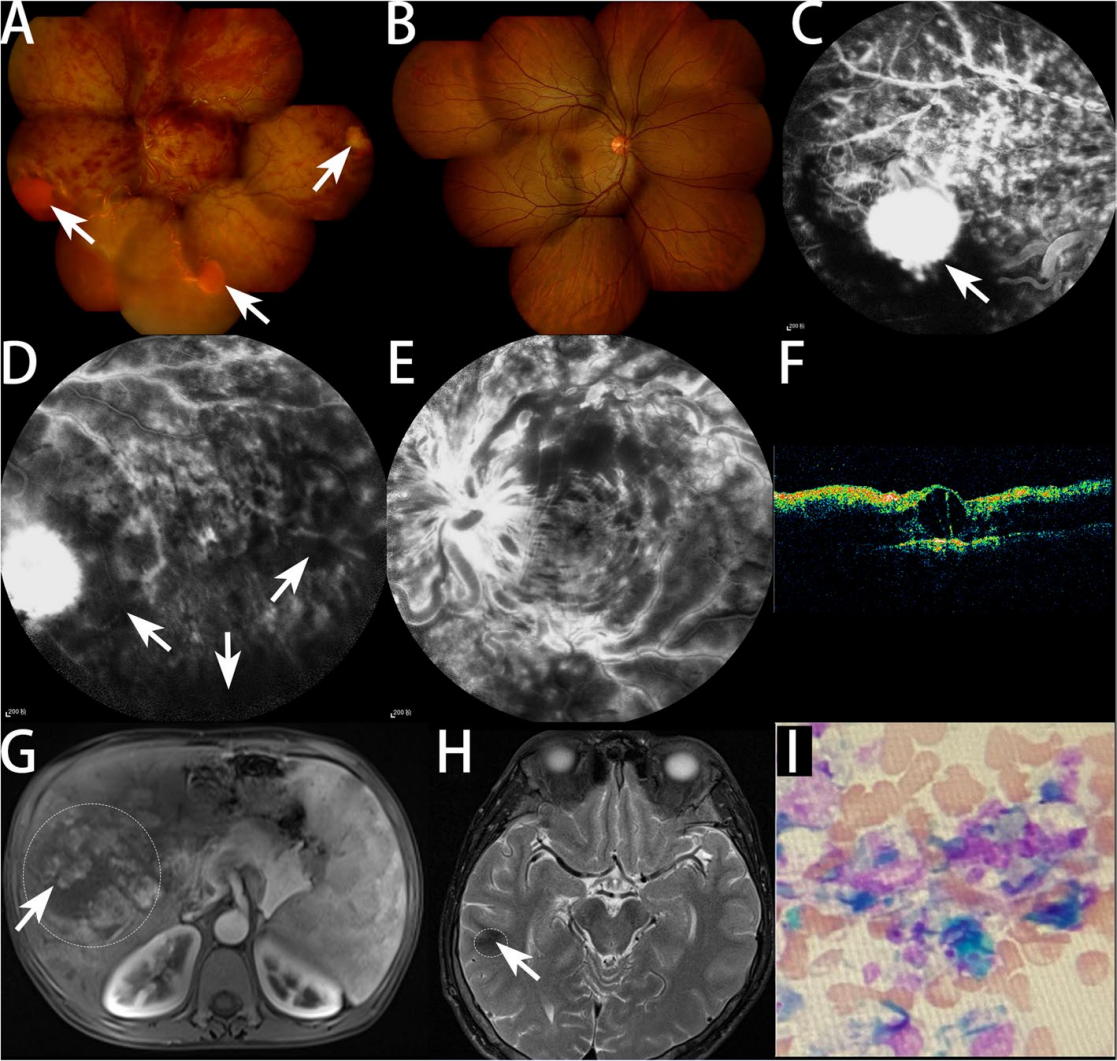
Clinical examination results of proband. **A** Color fundus image of proband's left eye. Diffuse patchy hemorrhages in the retina, and obvious earthworm-like tortuosity of the veins. Three retinal hemangioblastomas (white arrows) were found in the peripheral retina, all about 1 PD in size. **B** Color fundus image of proband's right eye. There is no obvious abnormality. **C** FFA image of proband's left eye. A high fluorescence lesion (white arrow) with the size of 1.5 PD were found. A thick and tortuous nourishing blood vessel is connected to it. Hemorrhage on the lower side of the lesion obscured fluorescence. Fluorescent leakage and staining of retinal veins. **D** FFA image of proband's left eye. Fluorescent leakage and staining of retinal veins. A high fluorescence lesion on the left side of the image. Non-perfusion areas were fund in the retina on the right and lower sides of the image (white arrows). **E** FFA late phase image. FFA of the left eye showed tortuous dilation of retinal vein, fluorescein staining of optic disc and retinal vein vessels, and flower-petal appearance of the leakage at the macula. **F** OCT image of proband's left eye. The retinal thickness in the macular area increased, and dark fluid-filled cyst inside the retina. **G** MRI image of proband's abdomen. A huge tumor (white arrow) was found in the right anterior lobe of liver with a size of 15.6 cm*11.4 cm, and the boundary with surrounding normal liver tissue was unclear (the white dotted line marks its approximate range). Snowflake enhancement was found in the tumor under enhanced scanning, and the spleen was obviously enlarged. **H** MRI image of proband's brain. A low signal area was found in the right temporal lobe with a size of 3 cm*2 cm (the white dotted line marks its approximate range, white arrow). **I** Bone marrow smear showed that nucleated cells proliferated actively, granulocyte: erythroid = 4.24:1, granulocyte: lobulated nuclear granulocyte ratio increased significantly, erythroid: proliferation was dominated by middle and late young erythrocytes, mature erythrocytes were distributed in accumulation, lymphocyte: ratio decreased, cell morphology was not significantly abnormal, combined with blood routine results: wbc12.99 × 109/L、RBC9.55 × 1012 / L, that is consistent with the diagnosis of polycythemia

**Fig. 2:**
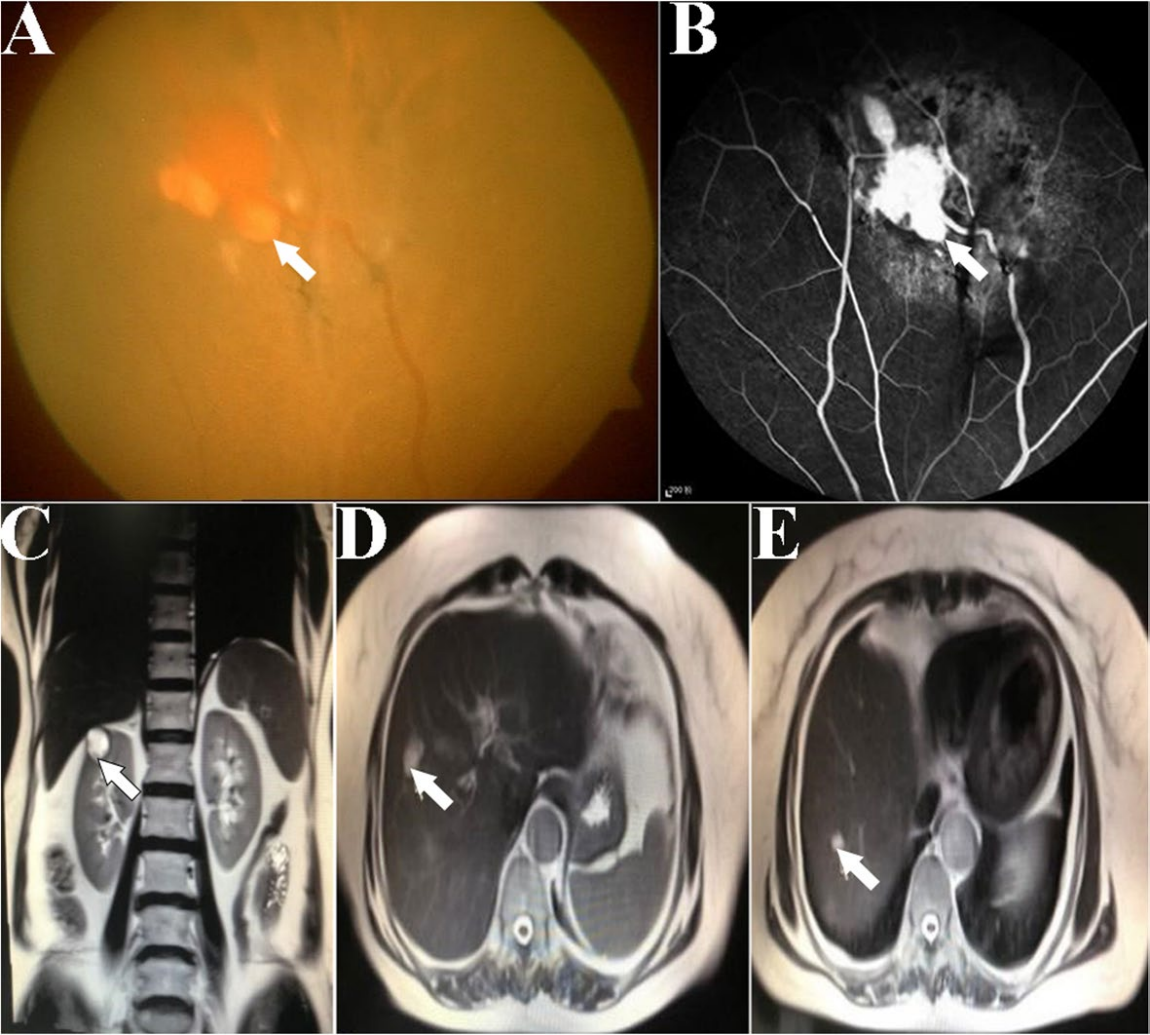
Clinical examination results of proband’s mother. **A** Color fundus image of I2's left eye. A yellow-white irregular lesion with a size of about 1/4 PD (white arrow) were found. **B** FFA image of patient I2's left eye. A high fluorescence lesion was found at the lesion corresponding to the Fig. 2A, and fluorescein leakage (white arrow) were found around the lesion. **C** T2 weighted image of I2's abdomen MRI showed a cystic lesion in the upper right kidney (red arrow). **D** and **E** T2 weighted images of I2's abdomen MRI showed two cystic lesions in the right anterior lobe of liver (white arrows)

A 20s male patient presented to our hospital and complained of a half-year vision loss with the left eye. His elder brother had undergone vitrectomy in both eyes for RHBs ten years ago. The physical examination showed that the best corrected visual acuity (BCVA) of left and right eyes was 0 (logMAR) and counting fingers (1m), respectively. Conjunctiva congestion in both eyes. The left fundus showed tortuous and dilated of retinal blood vessels, retinal flame-shaped hemorrhages, and 3 orange-red lesions in the peripheral retina (Fig. 1A). No obvious abnormality was found in the right fundus (Fig. 1B). The full physical examination revealed flushing of the skin, and eliminated language, smell and emotional barriers. Fundus fluorescein angiography (FFA) confirmed that the orange-red lesions of the retina were RHB (Fig. 1C), delay in retinal artery phase filling time, delay in retinal arteriovenous transit time and non-perfusion areas were found in the inferior peripheral retinal (Fig. 1D). Cystoid macular edema (CME) was confirmed by FFA (Fig. 1E) and optical coherence tomography (OCT, Fig. 1F). Magnetic resonance imaging (MRI) revealed a liver tumor and an old cerebral infarction in the right temporal lobe (Fig. 1G and H). Hematologic parameters were as follows: hemoglobin, 214.0 g/L; hematocrit, 72%; mean cell volume, 84.1 fL; RBC, 8.50 10^12^/L; WBC, 7.73 10^9^/L; and platelets, 226 10^9^/L. Bone marrow puncture smear showed the three main hematopoietic cell lines hyperplasia and accumulation like distribution of mature red blood cells, which was consistent with the characteristics of polycythemia (Fig. 1I). In the subsequent detailed family history investigation, it was found that the proband’s mother with RHBs (Fig. 2A and B), renal cyst (Fig. 2C) and liver cysts (Fig. 2D and E).

**Fig. 3:**
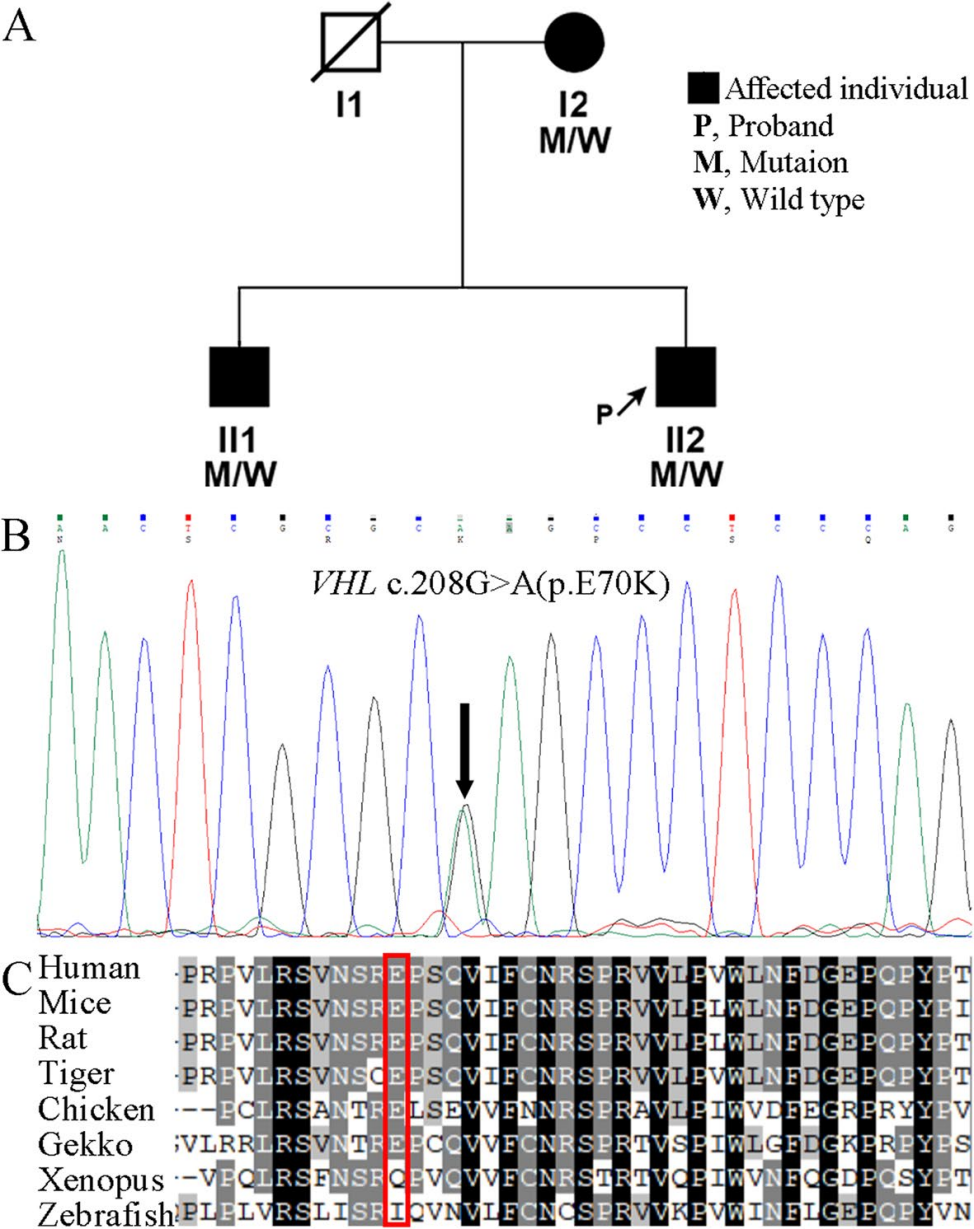
DNA analysis of the patients and family members. **A** The pedigree of the family with *VHL* c.208G > A. This family presents a co-segregation of genotypic phenotypes associated with *VHL* gene heterozygous mutation. **B** Sanger sequencing electropherogram showing compound heterozygous variant (black arrow) in *VHL*. **C** Conservation of E70 in VHL in different mammal, bird, and reptile species. The protein sequences of VHL orthologs at positions 59–100 are aligned. The red box indicates the position of E70

According to the Declaration of Helsinki, the patient and his family signed an informed consent form. Peripheral blood samples and clinical data of 3 individuals of the family were collected. Considering the unusual phenotypes and family medical history, whole-exome sequencing was performed for the proband. The results showed that the *VHL* c.208G > A (p.E70K) variant and the *EGLN1* c.380C > G (p.C127S) variant, while excluding *JAK2, EPOR, EPAS1, EPO, HBB, HBA1, HBA2, BPGM* and other gene mutations closely related to polycythemia. Sanger sequencing analysis of the family members revealed that both the mother and elder brother of the proband have the same genetic variants as him (Fig. 3A, B and C). In silico analysis indicated the pathogenic nature of the c.208G > A variant in *VHL* gene and the non-pathogenic nature of the c.380C > G variant in *EGLN1* gene. *VHL* c.208G > A has been predicted by Invitae as “likely pathogenic”, which replaces glutamic acid with lysine at codon 70 of the VHL protein and protein features might be affected. However, *EGLN1* c.380C > G has been predicted by Invitae as “benign”. In the 1000genomes database, the G allele frequency of this single nucleotide variant in East Asian population was 0.4593. And there was no report of the pathogenic case of *EGLN1* c.380C > G. Accordingly, it is revealed that the multi-organ hemangioblastoma and polycythemia in the proband are caused by *VHL* c.208G > A.

**Fig. 4:**
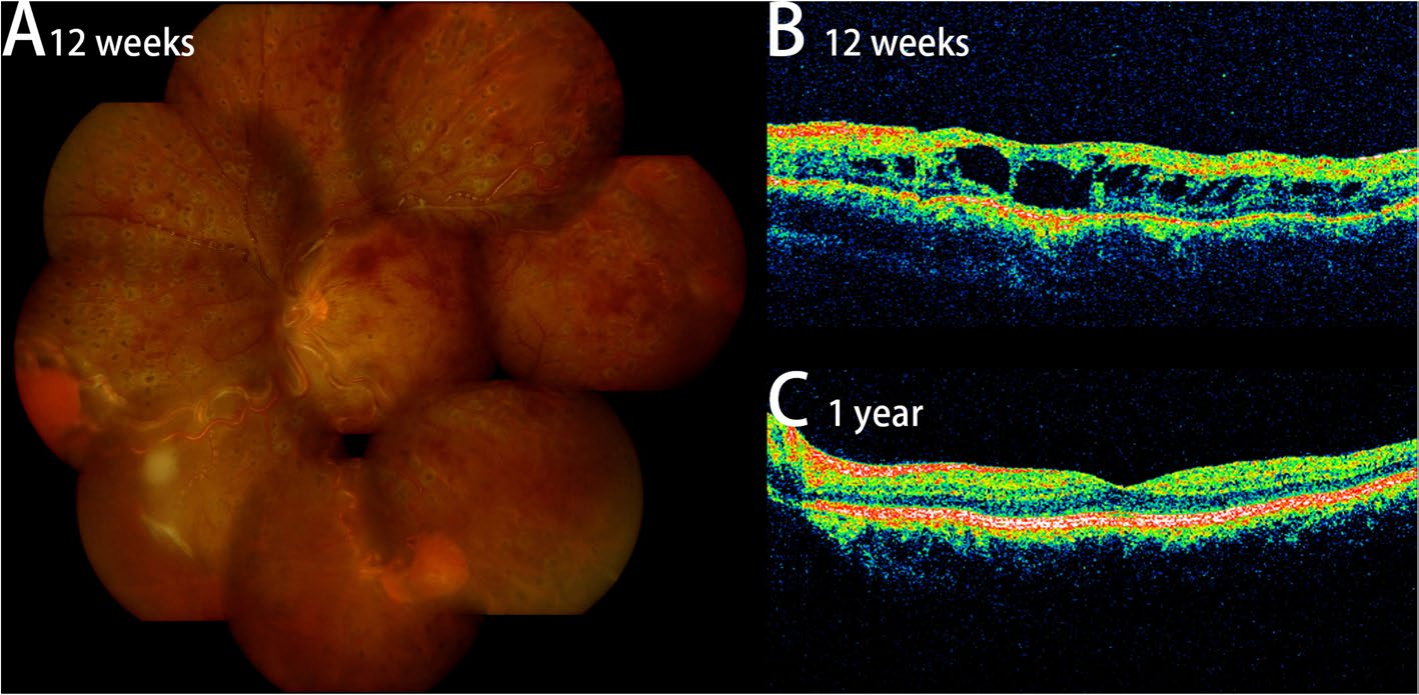
Results of proband after treatment. **A** Color fundus image of proband's left eye after 12 weeks. Most retinal hemorrhages were absorbed, and the tortuosity of retinal veins was partially relieved. Laser spots are widely distributed in the peripheral retina (**B**) OCT image of proband's left eye after 12 weeks. The retinal thickness in the macular region was not significantly reduced compared with that at the initial visit, and dark fluid-filled cyst inside the retina. **C** OCT image of proband's left eye after 1 year. The retinal thickness in the macular region was significantly reduced compared with that before, and macular edema was completely absorbed

Finally, the proband was diagnosed with VHL disease complicated with polycythemia and CRVO. As the patient with non-perfusion areas and CME, he was advised intravitreal anti-VEGF and a panretinal photocoagulation. He received panretinal photocoagulation (532 nm) in ophthalmology, and undergo phlebotomy regularly to maintain hematocrit < 45% in hematology. After 12 weeks, the patient's best corrected visual acuity improved to 1.3 (logMAR), most retinal hemorrhages were absorbed, and the tortuosity of retinal veins was relieved (Fig. 4A). No significant decrease in foveal thickness from baseline (Fig. 4B). Patient again refused intravitreal anti-VEFG, so macular grid laser photocoagulation (577 nm) was performed in the left eye. He insisted on undergo phlebotomies regularly in hematology department. After 1 year later, the macular edema in the left eye was absorbed (Fig. 4C) and the BCVA improved to 0.2 (logMAR).

## Discussion and conclusions

Here, we described a case of VHL disease with polycythemia and CRVO caused by *VHL* c.208G > A. The patient presented phenotypes including RHB, liver tumor, elevated hematocrit and CRVO. To the best of our knowledge, this is the first time that *VHL* c.208G > A has been reported in Chinese, and the first case report of *VHL* c.208G > A variant in the etiology of polycythemia and CRVO. And, RHB was found in all affected individuals in this family. In the past, the most of c.208G > A variant patients being reported were in South Korea [6–9]. Their clinical phenotypes include CHB, RHB, RCC and colorectal adenocarcinoma. The incidence of hemangioblastoma in patients with VHL disease caused by *VHL* c.208G > A was as high as 88.9%. CHB and RHB account for 50% and 38.9%, respectively (Table 1) [6–9]. The HIFα binding site is located at residues 65-117of pVHL. The possible pathogenic mechanism is that *VHL* c.208G > A (p.E70K) mutated the 70E of pVHL, which may affect the ubiquitination of HIFα. The dysregulated HIFα eventually leads to the occurrence of hemangioblastoma [10].

**Table 1:** Phenotypes of VHL disease caused by *VHL* c.208 g > A

Family	Gender	FH	CHB	RHB	Others	Reference
1	M	Proband	-	+	Polycythemia	-
	F	Mother	-	+	RC	
	M	Brother	-	+	-	
2	M	-	+	-	-	[6]
3	M	-	+	-	-	[7]
4	F	Proband	-	+	-	[7]
	M	Son	-	-	-	
5	F	Proband	-	+	-	[7]
6	M	-	+	-	-	[8]
7	M	-	+	-	-	[8]
8	M	-	+	X	X	[8]
9	M	-	-	+	-	[8]
10	F	-	+	-	Cs	[8]
11	F	-	+	-	Cs	[8]
12	F	-	-	+	-	[8]
13	F	-	+	-	-	[8]
14	M	Proband	+	X	RCC, CA	[9]
	M	Son	-	-	-	

*Abbreviations*: *M* Male, *F* Female, *FH* Family history, *CHB* Central nervous system hemangioblastoma, *RHB* Retinal hemangioblastoma, *RC* Renal cyst, *Cs* Pancreatic cyst, renal or hepatic cyst, *RCC* Renal cell carcinoma, *CA* Colorectal adenocarcinoma, *X* Data not available

Cases of polycythemia caused by mutations in the *VHL* gene are not common. According to the mechanism, these polycythemias are divided into two categories: one is secondary polycythemia caused by the secretion of erythropoietin (EPO) by renal cell carcinoma, cerebellar hemangioblastomas, and hepatocellular carcinoma of VHL disease; the other is the *VHL* gene mutation which changes the activity of pVHL and affects HIFα pathway increases EPO synthesis, resulting in erythrocytosis type 2 [11]. Patients with erythrocytosis type 2 either are carrying the homozygous state or compound heterozygous with the R200W mutation. In addition, VHL disease-related tumors were not found in patients with erythrocytosis type 2 [12]. EPO is synthesized and secreted by kidney (90%) and liver (10%), and reaches bone marrow through blood circulation to play a role in promoting the proliferation, differentiation and maturation of erythroid progenitor cells [11]. Therefore, when renal cell carcinoma, hepatocellular carcinoma, and the recently discovered cerebellar hemangioblastomas become additional sources of EPO, excessively high levels of EPO cause massive bone marrow erythroid hyperplasia, and eventually lead to polycythemia. Although the proband had no solid kidney lesion, but a huge tumor was found in his liver. His polycythemia is more likely to be caused by abnormal secretion of EPO from liver tumors. Unfortunately, the patient refused to accept pathological examination related to liver tumor and corresponding treatment.

As well known, CRVO is a common retinal vascular disease and a common loss of vision in older patients. The main risk factor for central retinal vein occlusion is age, 90% of patients are over 50 years old [13]. But this 20 s old patient also developed a rare CRVO in his left eye. The patient without small optic disc and juxtapapillary space-occupying lesions, thus excluded optic nerve hypoplasia and RHC as risk factors for CRVO. Further, after excluding other common CRVO risk factors such as hypertension, hyperlipidemia, diabetes, and retinal vascular inflammation, all the clues focused on polycythemia. Polycythemia is an uncommon predisposition for CRVO [13, 14]. It may be that a large number of circulating red blood cells lead to increased erythrocyte aggregation and blood hyper viscosity [14]. In addition, Lu/BCAM on the surface of erythrocytes was phosphorylated when polycythemia. Then erythrocytes and endothelial cells adhered due to the interaction between Lu/BCAM and laminin α5. This process simultaneously activates endothelial cells and stimulates the expression of vascular cell adhesion molecules, which is conducive to leukocyte adhesion [15]. Wautier MP et al. [16] also found a similar molecular mechanism in CRVO patients. The arm-choroid filling time was found to correlate with hematocrit level and platelet counts as the artery-venous transit time was found to correlate to the hematocrit and hemoglobin levels [17]. In a recent report, high blood viscosity and erythrocyte vascular adhesion caused by polycythemia can lead to delay in retinal arteriovenous transit time and retinal artery phase filling time, and finally lead to ischemic retinopathy [18]. In this patient the same phenomenon was observed, therefore ischemic lesions of the eye and brain may have been caused by blood hyper viscosity due to polycythemia. The difference is that the retinal ischemia caused by this factor is relatively mild, which is manifested as small patches of non-perfusion areas with indistinct borders. However, the mechanisms of CRVO caused by VHL disease could be diverse. In the report of AlBloushi AF et al. [19], a 22-year-old woman with VHL disease developed hemiretinal vein occlusion due to the mechanical compression of the juxtapapillary RCH. Our patient refused intravitreal anti-VEGF. CME existed for a long time, which can lead to loss of vision. Singh et al. [20] reported a case of BRVO caused by secondary erythrocytosis. A good visual acuity was restored after regular anti-VEGF and phlebotomy therapy.

In conclusion, we present a rare case of polycythemia complicated by CRVO in patient with VHL disease. It reminds us that the systemic disease factors should be fully considered in the diagnosis of young patients with CRVO, and that treatment requires a coordinated effort of physicians.

## Data Availability

The datasets used and/or analyzed during the current study are available from the corresponding author on reasonable request.

## Abbreviations

BPGM: Bisphosphoglycerate Mutase
CHB: Central nervous system hemangioblastoma
CME: Cystoid macular edema
CRVO: Central retinal vein occlusion
EGLN1: Egl-9 Family Hypoxia Inducible Factor 1
EPAS1: Endothelial PAS Domain Protein 1
EPO(R): Erythropoietin (Receptor)
FFA: Fundus fluorescein angiography
HBA: Hemoglobin Subunit Alpha
HBB: Hemoglobin Subunit Beta
HIFα: Hypoxia-inducible factor α
JAK2: Janus kinase 2
logMAR: Logarithm of the Minimum Angle of Resolution
MRI: Magnetic resonance imaging
OCT: Optical coherence tomography
RBC: Red blood cell
RCC: Renal cell carcinoma
RHB: Retinal hemangioblastoma
VHL: Von Hippel-Lindau
WBC: White blood cell
